# Maxillary Soft Tissue Mass in a Newborn Imposing as Teratoma: Diagnostic Challenges and Management Considerations

**DOI:** 10.7759/cureus.60631

**Published:** 2024-05-19

**Authors:** Shivani S Bothara, Pratapsingh Parihar, Shivali V Kashikar, Pratiksha Sachani, Ravishankar Patil

**Affiliations:** 1 Radiodiagnosis, Jawaharlal Nehru Medical College, Datta Meghe Institute of Higher Education and Research, Wardha, IND

**Keywords:** mature teratoma, nasolabial mass, surgical intervention, multidisciplinary approach, ct imaging, maxillary anomaly, neonate

## Abstract

Congenital facial teratomas in neonates pose diagnostic challenges, necessitating a multidisciplinary approach for accurate diagnosis and management. We present the case of a four-day-old female infant delivered via Lower Segment Cesarean Section (LSCS) with a protruding nasolabial mass noted since birth. CT brain plain revealed a soft tissue density opacification arising from the left maxilla with an underlying bony outgrowth, suggestive of a benign congenital developmental anomaly most likely teratoma. Further evaluation is warranted to delineate the exact nature and extent of the anomaly. This case underscores the importance of meticulous evaluation and interdisciplinary collaboration in managing congenital anomalies, with surgical intervention potentially required based on individual patient factors. Continued research and collaboration among medical specialities are essential to improve understanding and management strategies for congenital facial teratomas.

## Introduction

Congenital nasolabial masses are rare anomalies that present unique diagnostic and management challenges in neonates. While these masses can arise from various facial structures, those originating from the maxilla are uncommon [1]. Understanding the clinical presentation, diagnostic approach, and management options for such anomalies is essential for providing optimal care to affected newborns in view of nasal obstruction and cosmetic purposes, too. Congenital nasal masses can result from a diverse array of etiologies, including benign and malignant neoplasms, developmental anomalies, and vascular malformations [2]. Maxillary involvement in these masses adds complexity to their diagnosis and management due to the proximity of vital structures such as the nasal cavity, orbit, and cranial base. Differential diagnoses for nasal masses in neonates include congenital nasal pyriform aperture stenosis, encephaloceles, dermoids, teratomas, and hamartomas [3].

Diagnostic imaging is pivotal in evaluating congenital nasal masses, delineating anatomical structures, assessing mass characteristics, and identifying associated anomalies. Computed Tomography (CT) is often utilised for its ability to provide detailed anatomical information and assess bony involvement [4]. Magnetic Resonance Imaging (MRI) may also be employed to evaluate soft tissue components and vascular involvement [5]. The management of congenital nasolabial masses depends on factors such as the size, location, histological type, and associated complications. Small, asymptomatic masses may warrant observation, while larger or symptomatic masses may require surgical intervention. Surgical approaches vary and may include endoscopic resection, open surgical excision, or both [6]. In cases of congenital nasal masses with maxillary involvement, a multidisciplinary collaboration among paediatricians, otolaryngologists, radiologists, and pediatric surgeons is essential for accurate diagnosis and optimal management planning. Long-term follow-up is often necessary to monitor for recurrence, assess functional outcomes, and address potential complications.

## Case presentation

**Figure 1:**
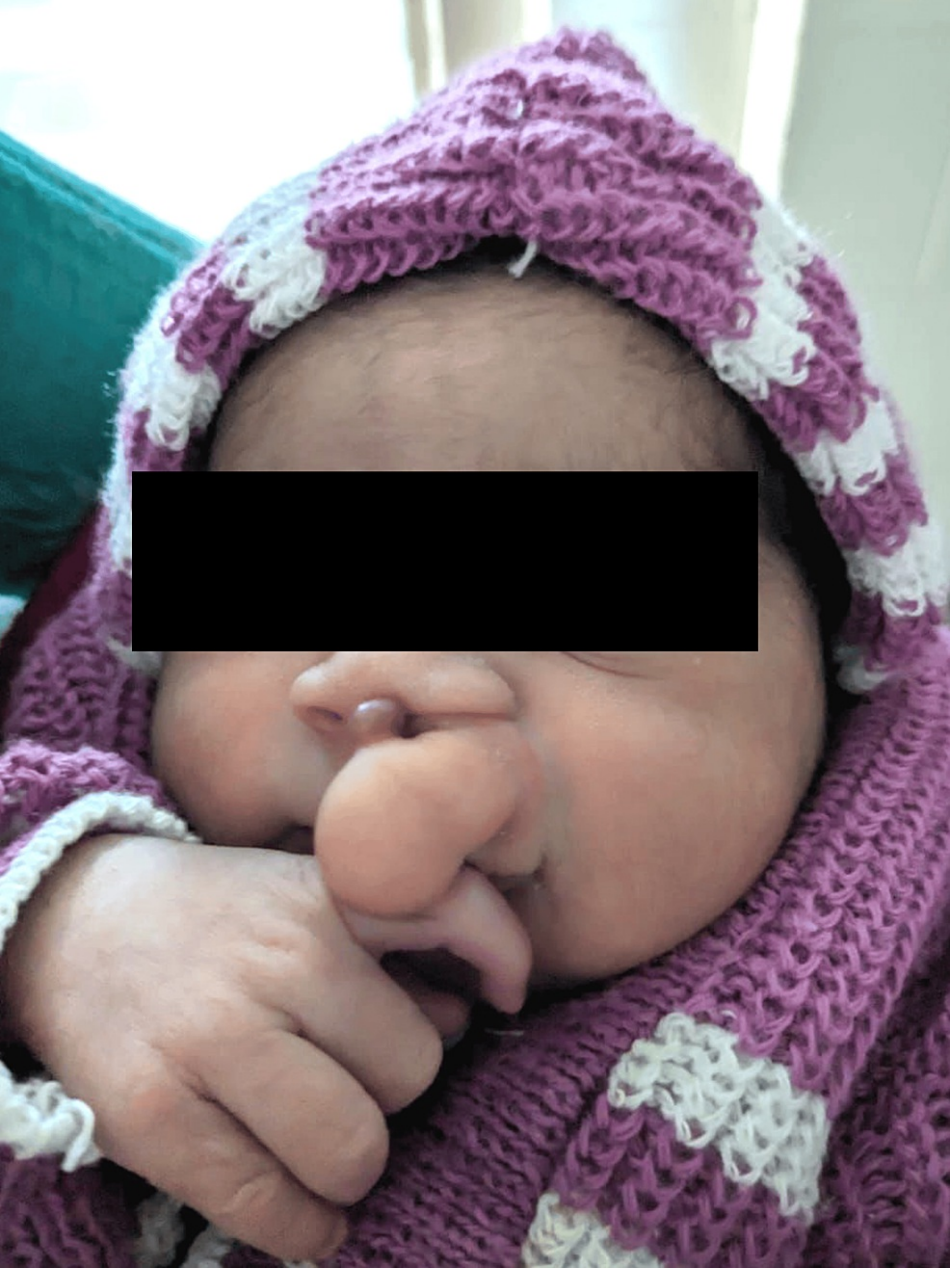
A clinical picture of the infant The image shows a protruding soft tissue hard mass in the left nasolabial region, partially obstructing the left nostril.

We present here a case of a four-day-old female infant, born via Lower Segment Cesarean Section (LSCS) on 18/3/24, presented with a protruding nasolabial mass since birth. Also, the lesion was present in her antenatal scans which were done outside. The patient's birth history indicated that she was the second gestation with one previous delivery (G2A1). Upon initial examination post-birth, a mass was observed protruding from the left nasolabial region obstructing the left nostril slightly Figure 1. Notably, no reported instances of nasal obstruction, breathing difficulty, or nasal discharge were reported. The anomaly had been diagnosed during the intrauterine period, and no other ear, nose, or throat (ENT) complaints were reported.

**Figure 2:**
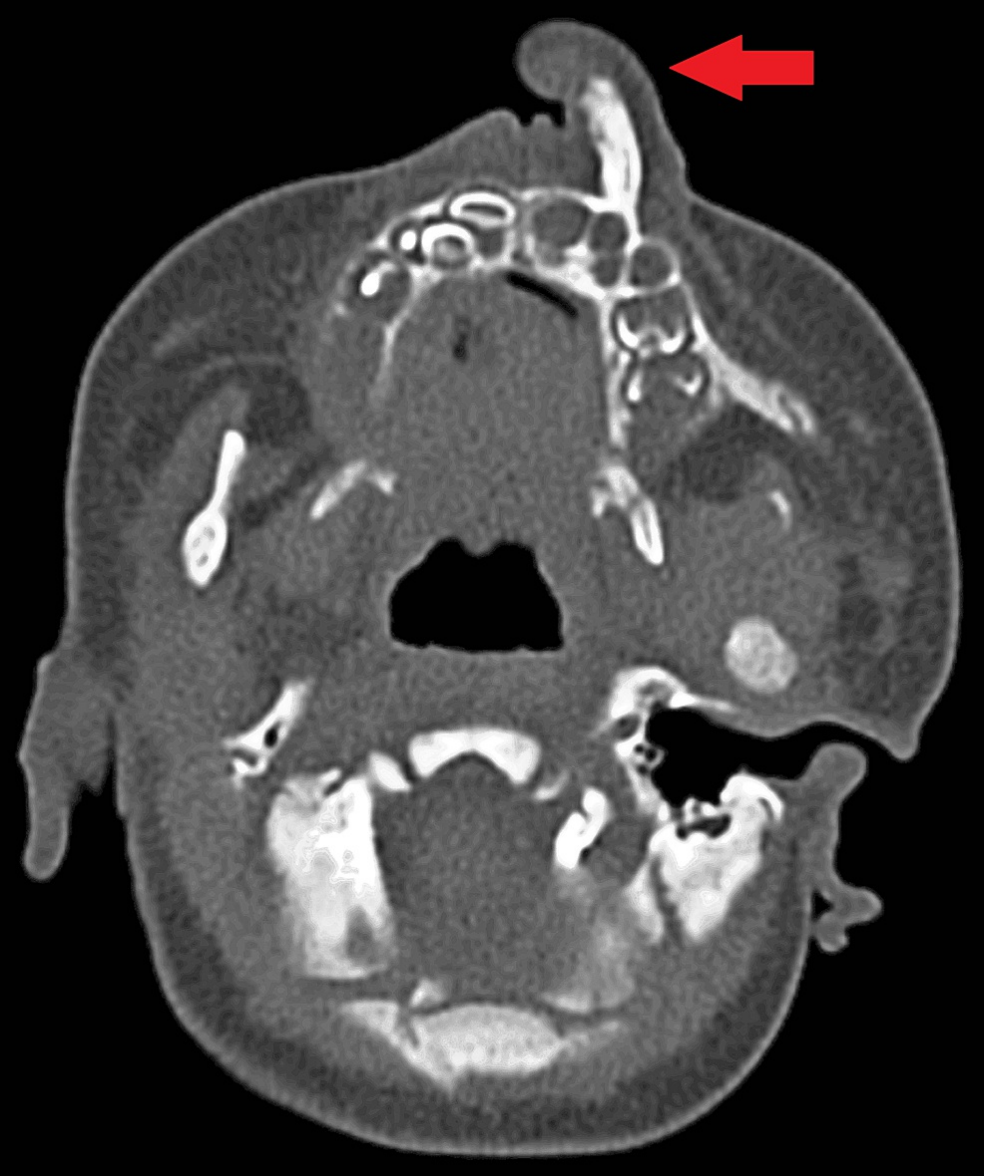
Non-contrast CT brain scan (bone window) The image shows a bony outgrowth protruding from the maxilla with adjacent soft tissue density mass surrounding it. The hard palate appears normal.

**Figure 3:**
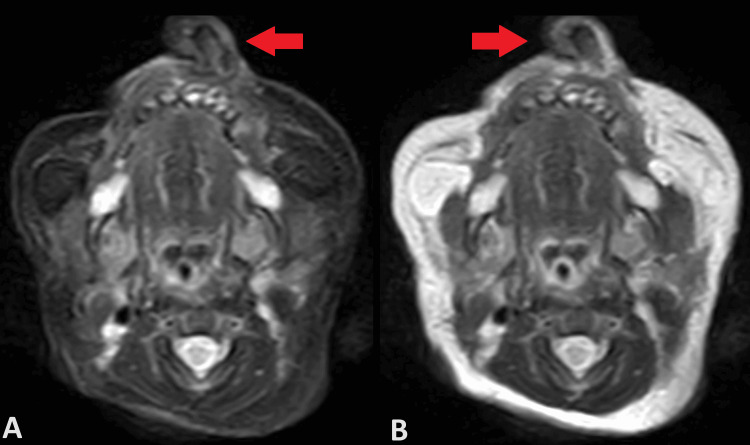
MRI brain plain (axial section) A) T2W fat suppressed scan image B) T2W image showing heterogenously enhancing soft tissue density lesion arising from maxilla with bony growth within. Features suggestive of teratoma.

Firstly, diagnostic imaging in CT brain plain was conducted to evaluate the condition further. The CT scan revealed several significant findings. Firstly, there was a well-defined soft tissue density opacification arising from the alveolar process of the left maxilla, measuring approximately 14 x 13 x 12 mm (Figure 2). An underlying bony outgrowth (spur) measuring approximately 14 mm from the left maxilla was also noted. Soft tissue density opacification was also observed in the left nasal cavity. Furthermore, an MRI of the brain plane was done, which showed hypertrophy of the left middle and inferior turbinate (Figure 3). However, cerebral parenchyma, gangliocapsular region, cerebellum, brainstem, cisternal spaces, ventricular system, and posterior fossa structures appeared normal. There was no connection with the brain parenchyma. The visualized orbit also displayed no abnormalities.

Based on these findings, the impression drawn from the CT brain was of a well-defined soft tissue density opacification arising from the left maxilla with an underlying bony outgrowth. The nature of this anomaly was indicative of a benign congenital developmental anomaly, most likely teratoma, necessitating further evaluation to determine its exact nature and extent.

Discussion surrounding this case emphasizes the complexity of diagnosing and managing congenital nasal masses with maxillary involvement. While the absence of significant airway obstruction or respiratory distress is reassuring, it is imperative to conduct a thorough evaluation to ensure appropriate management planning. Multidisciplinary collaboration involving pediatricians, otolaryngologists, and radiologists is essential for accurate diagnosis and optimal patient care. Further investigations, such as histopathological examination or additional imaging modalities, may be warranted to delineate the precise nature and extent of the anomaly.

## Discussion

The presented case highlights the diagnostic challenges and management considerations associated with congenital nasal masses involving maxillary structures in neonates. While such anomalies are rare, they require a comprehensive approach for accurate diagnosis and appropriate management to prevent potential complications and ensure optimal outcomes for the patient. The differential diagnosis for a neonate presenting with a nasal mass includes a wide range of possibilities, including congenital anomalies such as nasal glioma, encephalocele, teratoma, dermoid cyst, and benign soft tissue tumours [7]. In this case, the CT brain plain findings of a soft tissue density opacification arising from the left maxilla with an underlying bony outgrowth supported the diagnosis of a teratoma. However, further evaluation with histopathological examination is warranted to define the nature and extent of the anomaly precisely.

Imaging modalities play a crucial role in the evaluation of congenital nasal and nasolabial masses. CT imaging provides detailed anatomical information, allowing for the assessment of soft tissue and bony structures involved [8]. In our case, CT and MRI brain plain revealed the precise location and characteristics of the mass, aiding in diagnosis and treatment planning. However, it is essential to consider radiation exposure, especially in neonates, and balance the diagnostic benefits with potential risks.

Management of congenital nasal masses often involves a multidisciplinary approach. Pediatricians, otolaryngologists, radiologists, and sometimes neurosurgeons collaborate to formulate an appropriate management plan tailored to the individual patient's needs [9]. In this case, further evaluation, possibly including histopathological examination or additional imaging studies, may be necessary to determine the exact nature of the anomaly and guide treatment decisions. Surgical intervention may be indicated depending on the nature and extent of the anomaly. The goals of surgery include complete excision of the mass while preserving surrounding structures and minimising morbidity [10]. Long-term follow-up is essential to monitor for recurrence and ensure optimal postoperative outcomes.

## Conclusions

In conclusion, the presented case underscores the complexity and importance of a multidisciplinary approach in managing congenital nasolabial masses with maxillary involvement in neonates. Through detailed imaging modalities such as CT and MRI, the precise characterisation of the anomaly aids in formulating appropriate management strategies. Surgical intervention may be necessary, with the goal of complete excision while preserving surrounding structures and minimising morbidity. Long-term follow-up is essential to monitor for recurrence and ensure optimal postoperative outcomes. Continued collaboration among medical specialities and further research is imperative to enhance our understanding and management of these rare congenital anomalies, ultimately improving patient care and outcomes.
